# Clustering the Depression Prevalence in Indonesia Provinces through Natural Breaks Jenks Method

**DOI:** 10.2174/0117450179375982250512114928

**Published:** 2025-05-21

**Authors:** Widya Saputri Agustin, Herlin Ari Prastika, Gading Kaila Kendrasti, Rohmatul Fajriyah, Vang Le-Quy

**Affiliations:** 1 Study Program in Statistics, Universitas Islam Indonesia, Yogyakarta, Indonesia; 2 Master Program in Statistics, Universitas Islam Indonesia, Yogyakarta, Indonesia; 3 Novodan, Herningvej 296, Aalborg East 9220, Denmark

**Keywords:** Clustering, Natural breaks, Jenks method, Depression, Mental health, Indonesia

## Abstract

**Introduction:**

Depression is a major public health issue worldwide, ranking fourth among global diseases in 2022 according to the WHO. In Indonesia, the 2018 Basic Health Research (Riskesdas) reported that over 12 million individuals aged 15 and above suffer from depression. Identifying regional disparities in depression prevalence is essential to guide targeted mental health policies and interventions.

**Methods:**

This study employed the Natural Breaks Jenks classification to cluster depression prevalence across Indonesian provinces using data from the 2023 Indonesia Health Survey. This method effectively grouped provinces based on natural data patterns, enabling the identification of regions with low, medium, high, and very high depression prevalence.

**Results:**

The analysis revealed significant regional disparities. Eighteen provinces, including Papua, Maluku, and several Sulawesi regions, were classified as having low depression prevalence. Eleven provinces, such as Aceh, Bali, and Kalimantan Timur, fell into the medium category. Six provinces—including DKI Jakarta, Banten, and Sumatera Selatan—exhibited high prevalence rates, possibly due to urbanization and socio-economic factors. Critically, Jawa Barat, Jawa Tengah, and Jawa Timur were identified as having very high depression prevalence, suggesting urgent needs for intervention.

**Discussion:**

These findings underscore the need for geographically targeted mental health strategies. Provinces with very high prevalence require prioritized attention for mental health services, infrastructure, and resource allocation. Understanding local socio-economic and cultural contexts will be crucial in reducing disparities and improving national mental health outcomes.

**Conclusion:**

These results indicate that Indonesia has a higher number of provinces with low depression prevalence compared to those with high prevalence. This suggests that while there are regions with lower rates of depression, there are still significant areas where mental health issues need more focused attention. Given this, the government should prioritize provinces with very high depression prevalence to improve mental health outcomes in those areas. By focusing on these regions, the government can better allocate resources, implement targeted interventions, and provide necessary mental health services. Addressing the mental health needs of provinces with high depression rates is essential for reducing overall national mental health disparities and ensuring equitable access to mental health support across Indonesia. Additionally, understanding the socio-economic and cultural factors that contribute to higher depression rates in these regions will be crucial in designing effective and sustainable mental health programs.

## 1 INTRODUCTION

Depression is one of the most prevalent and severe mental health disorders globally, affecting millions of people across all continents, cultures, and age groups. The World Health Organization (WHO) has highlighted depression as a leading cause of disability worldwide and an important issue in global health. The condition is not only a challenge for individuals but also for societies, as it affects personal well-being, productivity, and the economy at large.

The World Health Organization (WHO) reported that over 264 million people worldwide suffer from depression [1]. It is a major contributor to the global disease burden, ranking as the leading cause of disability. Depression is a significant global health concern, ranking fourth among the world's diseases in 2022, with 55% of those affected experiencing suicidal thoughts. Depression’s widespread prevalence affects individuals across various demographics, with a particularly high incidence observed in women and adolescents. Depression is closely linked with suicide, which accounts for more than 700,000 deaths annually [1]. This highlights the critical importance of addressing depression as a global health issue, given its potential to escalate into more severe outcomes when left untreated.

Depression is a clinical syndrome. The International Classification of Diseases (ICD) diagnostic classification systems describe three core symptoms of depression: low mood, anhedonia, and reduced energy levels. Other symptoms include impaired concentration, loss of confidence, suicidal ideation, disturbances in sleep, and changes in appetite. Symptoms must have been present for at least two weeks for a diagnosis of depression to be made. Depressive symptoms, which can be clinically significant, can be present in the absence of a major depressive episode [2]

Depression is one of the most diagnosed mental disorders among adults. Our understanding of the course and nature of depression has changed significantly in the last 20 years. Depression is increasingly recognized not just as an acute, self-limiting illness, but as a chronic, lifelong condition for many individuals. The high prevalence of depression is concerning due to its substantial economic and social costs. It is economically detrimental and engenders significant personal and interpersonal suffering alongside its societal impact [3]

Depression is a disrupted state of human function related to feelings of sadness and accompanying symptoms, including changes in sleep and appetite patterns, psychomotor changes, concentration difficulties, anhedonia, fatigue, feelings of hopelessness and helplessness, and suicide [4]

Depression disorder is a mood disorder that comes in different forms. The three most common types of depression disorder are discussed below. There are also differences in how individuals experience depression based on age [5].

### 1.1 Mild Depression

A depressive episode must have at least two of the three main symptoms plus two of the other symptoms. The duration of the depressing episode is less than two weeks, and there is some difficulty in work and daily activities [6].

### 1.2 Moderate Depression

A depressive episode must have at least two of the three main symptoms plus three/four of the other symptoms. The duration of the depressive episode is at least two weeks, and there is significant difficulty in work and daily activities [6].

### 1.3 Severe Depression

Severe depression is characterized by a combination of symptoms that persist for at least two consecutive weeks, including a sad mood and or easily irritable mood, which can interfere with the ability to work, sleep, eat, and enjoy daily activities. Difficulty in sleeping or eating may be a contributing factor. This depressive episode can occur once, twice, or multiple times throughout one's lifetime [5].

In their research, the authors identified several factors that can influence depression, including health, personality, religiosity life experiences, bitterness, self-esteem, and social support [7]. Several experts also provided explanations regarding the cause of depression. The causal factors of depression include biological, genetic, and psychosocial factors, all of which can interact and influence one another.

The study highlighted several factors associated with youth depression, including being female, smoking, alcohol consumption, having a chronic disease, and having a parent with depression [8]. Common symptoms found in depressed adolescents include sadness, irritability, negative thinking, feeling worthless, poor school or college attendance, frequent misunderstandings or being overly sensitive, using drugs and alcohol, excessive eating or sleeping, self-harm, decreased interest in sports and hobbies, and distancing oneself from social environments [9].

Depression diagnosis is established if there are five or more of the following symptoms: significant changes in appetite, weight loss or gain, insomnia or hypersomnia, always feeling tired, feeling worthless, decreased concentration and memory disturbances, and thoughts of ending life. These symptoms must be experienced for at least 2 weeks or more, and feeling sad all the time [10].

In Indonesia, depression is a growing mental health concern with significant implications for both individuals and society. The Riskesdas 2018 [11] stated that the prevalence of depression among adults (aged 15 and older) was found to be approximately 6.1%. This means that around 12 million people in Indonesia are experiencing some form of depression. Furthermore, it was reported that depression is more common among women compared to men, where the prevalence of depression was 8.4% among women and 3.6% among men. The prevalence of depression was found to be higher in urban areas (6.5%) compared to rural areas (5.4%), and it was more prevalent among older adults, especially those aged 45 and above.

The Indonesia Ministry of Health has focused on preventive efforts to address mental health issues and collaborated across programs, one of which is immunization. Unlike general immunization, the mental health immunization program developed by the Directorate of P2MKJN is specifically for mental health. Its goal is to develop Indonesian human resources that are resilient, superior, strong, and immune to the rapid changes of the times [12].

While there have been several studies on the prevalence of depression in Indonesia, they typically do not explore the spatial variability or regional clustering of depression cases. There is limited research on how depression is distributed geographically across urban and rural areas and whether there are significant regional disparities that could inform targeted interventions.

This study aims to fill this gap by utilizing geospatial analysis to examine the spatial distribution and clustering of depression across Indonesia. The RStudio and QGIS software will be used to perform clustering on the prevalence of depression in Indonesia, by using the Natural Breaks Jenks method. The Natural Breaks Jenks method is a common technique used for clustering data presented in a Geographic Information System (GIS) product map [13, 14].

Given Indonesia’s vast geographic and socio-economic diversity, it is expected that depression prevalence may not be evenly distributed across the country, and certain areas may exhibit higher concentrations of depression.

By identifying regions with high depression rates, understanding the underlying spatial patterns, and tailoring interventions to meet local needs, policymakers can more effectively address the mental health crisis. This approach allows for targeted resource allocation, early intervention, and the promotion of health equity across diverse regions. Additionally, the lessons learned from Indonesia’s experience can inform mental health strategies in other countries facing similar challenges, ultimately contributing to global mental health advancements.

These limitations should be taken into account when interpreting the study's findings. Future research could address these constraints by using longitudinal data, incorporating more comprehensive socioeconomic and healthcare variables, and considering alternative diagnostic tools that are culturally appropriate for diverse Indonesian populations. Nonetheless, the current study provides valuable insights into regional disparities in depression prevalence and offers important implications for mental health policy and interventions in Indonesia.

## 2 METHODOLOGY

### 2.1 Data

The data used in this study are secondary data obtained from the 2023 Indonesian Health Survey on the prevalence of depression in the last two weeks among residents aged ≥15 years, which can be accessed at www.badankebijakan.kemkes.go.id/hasil-ski-2023. The assessment of depression in the 2023 Indonesian Health Survey (SKI) used the Mini International Neuropsychiatric Interview (MINI), which consists of 10 questions with “yes” or “no” answer options.

The questions were asked by the interviewer to individuals aged 15 years and older, and did not specifically represent the condition of the last two weeks. Respondents were categorized as experiencing depression if they answered “Yes” to at least 2 of the 3 main questions (questions 1-3) and “Yes” to at least 2 of the 7 additional questions (questions 4-10). The prevalence of depression in the last two weeks among residents aged ≥15 years was calculated using the formula (1) [14].


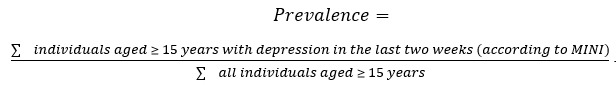
(1)

The data was collected by local enumerators under the technical supervision of District/City PJTs and administrative supervision by District/City PJOs. Each team was responsible for 10 to 12 census blocks (CB). Each CB consisted of 10 regular households, with an additional ± 7 households with children under five, to collect data on the nutritional status of children in that age group. Data collection began with coordination between the district PJT and the district PJO to identify sample locations. Based on this identification, enumerators were expected to get an overview of the sample locations so that they could plan an efficient and effective data collection schedule and strategy.

Before data collection, the SKI Household update process was conducted, where enumerators visited households listed in the Updated Household List (DPRT) obtained from BPS to verify the existence of these households and register any new households not previously listed. From the results of this update, samples of regular households, households with children under five, and overlapping households were selected for further data collection.

The data collection for the 2023 SKI was conducted through interviews, measurements, and examinations.

Out of the 34,500 selected census blocks (CB) for the 2023 SKI sample, 34,065 CB (98.74%) were successfully identified and visited through the data updating process, distributed across 38 provinces and 514 districts/cities. However, data could not be collected in 435 CBs (1.26%) in 7 provinces, namely North Sumatra, West Sumatra, North Kalimantan, West Papua, Papua, Central Papua, and Highland Papua, due to security stability conditions and adverse weather. The number of surveyed households is 315,646 (91.49%) out of the target of 345,000 regular households.

In total, there are 630,627 prevalence of depression in the past two weeks among individuals aged ≥15 by province in SKI 2023. The distribution for each province can be seen in Table **1**.

### 2.2 Limitation of the Study

While this study provides valuable insights into the regional disparities in depression prevalence across Indonesia, several limitations must be acknowledged, which could affect the interpretation and generalizability of the findings:

#### 2.2.1 Cross-sectional Nature of the Data

The data used in this study are cross-sectional, meaning they capture a snapshot of depression prevalence at a single point in time. This limits the ability to draw conclusions about causality or to track changes in depression rates over time. Longitudinal studies would be necessary to identify trends and better understand the causal relationships between regional factors and depression prevalence.

#### 2.2.2 Potential Response Bias

The data were obtained through the Mini International Neuropsychiatric Interview (MINI), which relies on self-reporting by individuals. This introduces the possibility of response bias, where participants may underreport or overreport their symptoms due to factors such as stigma surrounding mental health, personal perception of their condition, or lack of awareness about depression. Such biases could lead to either an underestimation or overestimation of depression prevalence in certain regions.

#### 2.2.3 Cultural Sensitivity of Diagnostic Tool

While the MINI is a validated tool for assessing mental disorders, it was primarily developed in Western contexts. There may be cultural differences in how depression symptoms are expressed or recognized, particularly in diverse Indonesian populations. This cultural variance could potentially influence how depression is diagnosed and reported in certain regions, particularly in rural or indigenous areas where the stigma associated with mental health may be higher.

#### 2.2.4 Geographical and Socioeconomic Factors

Although this study identifies regional disparities, it does not fully account for all possible socioeconomic and environmental factors that may influence depression rates, such as income inequality, unemployment rates, education levels, or access to healthcare. A more thorough exploration of how these variables intersect with depression prevalence could provide deeper insights into the root causes of regional differences.

#### 2.2.5 Healthcare Infrastructure and Access

The study did not directly measure the availability or quality of mental health services in each province. Provinces with higher depression rates may have more accessible healthcare services, resulting in higher reported prevalence rates, whereas those with fewer mental health resources may underreport due to lack of awareness or access. This uneven distribution of mental health services across regions could skew the interpretation of depression prevalence and limit the generalizability of the findings to regions with varying healthcare infrastructure.

#### 2.2.6 Exclusion of Certain Demographic Groups

The study focuses on individuals aged 15 and above, and the exclusion of children and adolescents may limit the comprehensiveness of the findings, particularly considering that early onset of depression can significantly affect long-term mental health outcomes. Including a wider age range would provide a more holistic view of depression prevalence across all age groups.

#### 2.2.7 Data Quality and Reliability

Although the 2023 Indonesian Health Survey and the use of MINI are reliable sources, there may be errors in data collection, such as misclassification of depression status, inconsistent interview techniques, or challenges in conducting interviews in remote areas. These potential sources of error could impact the reliability and accuracy of the reported depression rates.

### 2.3 Natural Breaks Jenks Clustering

Clustering is a fundamental data analysis method [15]. It is widely used for pattern recognition, feature extraction, vector quantization (VQ), image segmentation, function approximation, and data mining. As an unsupervised classification technique, clustering identifies some inherent structures present in a set of objects based on a similarity measure.

There are several clustering methods, one of which is the natural breaks method. Natural breaks clustering identifies groups based on patterns or groupings that naturally occur within the data. Cluster breaks are identified as the best group with similar values and that maximize the differences between clusters. Features are divided into clusters, with boundaries placed where there are relatively large differences in data values. The Jenks optimization method, also called the Jenks Natural Breaks clustering method, is a data clustering method designed to determine the best arrangement of values into different clusters [13]. The Natural Breaks Jenks method is based on the Natural Breaks Jenks algorithm [16]. This method seeks to minimize the average cluster deviation from the cluster meanwhile maximizing the average cluster deviation of the means of other groups [16]. Therefore, variances are minimized within the clusters but maximized between the clusters.

The Natural Breaks Jenks method’s purpose is to display a set of clustering values that most accurately represent the actual breaks observed in the data compared to other clustering schemes [17]. This method aims to identify patterns and divide spatial data into homogeneous groups [18]. The Natural Breaks Jenks method plays a crucial role in mapping analysis, such as geospatial analysis, regional cluster analysis, or statistical data mapping. This method helps in understanding the patterns and characteristics of depression prevalence across provinces in Indonesia, which are related to spatial entities in the data, as well as facilitating decision-making based on the results of clustering.

This paper implemented the Natural Breaks Jenks method using RStudio and QGIS by following the work of [19], where the prevalence of depression by province was divided into 4 clusters according to [20]. The 4 clusters consist of: “low”, “medium”, “high”, and “very high” cluster categories. Building on previous studies [19], the steps for performing Natural Breaks Jenks clustering in this research are as follows:

- Set the working directory by clearing the workspace (removing all unnecessary datasets and variables) and closing any previously created graphs.
- Load the required packages such as **dplyr**, **ggplot2**, and **classInt**.
- Read the data to be analyzed using the **read.csv()** function since the dataset file is in CSV format.
- Use the **classIntervals()** function to compute natural breaks using the Jenks method and specify the number of clusters (n) as 4.
- Save the cluster boundaries generated from the natural breaks calculation.
- Create a data frame containing the data to be analyzed.
- Use the **cut()** function to classify the data into clusters based on the specified cluster boundaries.
- Utilize the **group_by()** and **summarize()** functions from the **dplyr** package to calculate the number of observations in each cluster.
- Generate a plot using **ggplot2** using a dot plot and customize the axis labels and plot title.
- Determine the clustering labels as “low”, “medium”, “high”, and “very high” according to the specified cluster boundaries.
- Mapping cluster of depression prevalence in Indonesia using QGIS.

The Natural Breaks Jenks method is well-suited for clustering depression prevalence in Indonesia due to its ability to detect natural groupings in the data, its flexibility in determining the number of clusters, and its appropriateness for datasets with skewed distributions. It allows for meaningful and interpretative divisions that align with the geographic and social realities of depression prevalence across Indonesia. In contrast, methods like k-means or hierarchical clustering may impose artificial structures on the data that do not accurately reflect the underlying geographic or socioeconomic variations in depression rates. Therefore, the choice of the Jenks method is not only methodologically sound but also enhances the interpretability and policy relevance of the findings.

## 3 RESULTS

### 3.1 Data Description

Fig. (**1**) shows the prevalence of depression over the last two weeks among residents aged ≥15 in 38 provinces across Indonesia, based on the Indonesian Health Survey 2023. The figure reveals that the three provinces with the highest depression prevalence are located on Java Island. In contrast, DIY (Yogyakarta) province, known for being a student city with a peaceful environment, exhibits the lowest depression prevalence on Java Island. The three provinces with the lowest depression prevalence in Indonesia are located on Papua Island, namely Papua Barat Daya, Papua Barat, and Papua Selatan.

### 3.2 Clustering Result

The Natural Breaks (Jenks) calculation divided the dataset into four distinct clusters, each representing a specific range of values along with the corresponding number of observations. The first cluster (low) includes values from 0,982 to 6,832 and consists of 18 observations. The second cluster (medium) ranges from 6,833 to 14,408, with 11 observations. The third cluster (high) covers values from 14,409 to 33,667 and contains 6 observations. Finally, the fourth cluster (very high) spans from 33,668 to 113,568, with only 3 observations Table **2**.

These divisions are determined by the Jenks method, which optimizes the internal variation within clusters while maximizing the external variation between them. As a result, this partitioning facilitates a clear distinction between different clusters within the dataset based on their observed values. The numerical statistical descriptives of mean, median, and standard deviation for each cluster support this claim.

The results of dividing depression prevalence clusters by province using the natural breaks (Jenks) method in RStudio are shown in the bar plot in Fig. (**2**).

Fig. (**3**) is a visualization of the distribution of depression prevalence clusters in the last 2 weeks in residents aged ≥15 years according to province using QGIS software. Provinces colored blue are included in the “low” cluster, provinces colored green are included in the “medium” cluster, provinces colored yellow are included in the “high” cluster, and provinces colored red are included in the “very high” cluster.

Based on four categories which are low, medium, high, and very high. The survey revealed significant regional disparities in depression prevalence across Indonesia's provinces. Low prevalence was found in 18 provinces: Bengkulu, Kepulauan Bangka Belitung, Kepulauan Riau, Kalimantan Tengah, Kalimantan Utara, Sulawesi Utara, Sulawesi Tenggara, Gorontalo, Sulawesi Barat, Maluku, Maluku Utara, Papua Barat, Papua Barat Daya, Papua, Papua Selatan, Papua Tengah, and Papua Pegunungan. Medium prevalence occurred in 11 provinces: Aceh, Sumatera Barat, Riau, Jambi, DI Yogyakarta, Bali, Nusa Tenggara Barat, Nusa Tenggara Timur, Kalimantan Barat, Kalimantan Selatan, and Kalimantan Timur. High prevalence was observed in 6 provinces: Sumatera Utara, Sumatera Selatan, Lampung, DKI Jakarta, Banten, and Sulawesi Selatan. A very high prevalence was found in 3 provinces: Jawa Barat, Jawa Tengah, and Jawa Timur.

The Kruskal-Wallis test was followed by the Tukey HSD posthoc test, with alpha 0.05, revealing that there are statistically significant differences in depression prevalence across provinces in Indonesia based on those four clusters.

## 4 DISCUSSION

The application of the Natural Breaks (Jenks) method to analyze depression prevalence across Indonesia’s provinces provides valuable insights into the geographic distribution of this mental health condition. By clustering provinces into four distinct groups based on depression prevalence rates, a clearer understanding of regional disparities in mental health has emerged.

In the low prevalence cluster, which includes 18 provinces, depression rates ranged from 982 to 6,832, reflecting relatively lower levels of depressive symptoms. All provinces in Papua Island show the lowest depression prevalence, suggesting that geographical location may influence mental health trends. Exploring the socio-cultural dynamics, access to mental health services, and community support systems in these regions could reveal protective factors that contribute to these lower rates. Furthermore, this low prevalence may indicate fewer mental health-related resources or less awareness of mental health issues in these provinces, which could be further examined for better service allocation.

The medium prevalence cluster consists of 11 provinces, with depression rates ranging from 6,833 to 14,408, reflecting moderate levels of depressive symptoms. Understanding the factors contributing to depression in these areas, such as economic stability, social cohesion, and local cultural attitudes toward mental health, could inform the development of targeted prevention and early intervention strategies. Tailored community outreach programs and education initiatives can help reduce stigma and encourage individuals to seek help at the primary care level.

In the high prevalence cluster, six provinces exhibited depression rates ranging from 14,409 to 33,667, indicating elevated levels of depressive symptoms. The contributing factors in these regions may include urbanization, socio-economic disparities, and limited access to mental health services. The increasing pressures associated with urban living, such as stress, isolation, and economic challenges, might exacerbate mental health issues in these regions. Therefore, comprehensive public health strategies that focus on providing mental health services, improving awareness, and addressing socio-economic inequalities are crucial to mitigating these disparities.

Finally, the very high prevalence cluster, which includes Jawa Barat, Jawa Tengah, and Jawa Timur, displayed depression rates ranging from 33,668 to 113,568. These areas show the highest levels of depression, signaling an urgent need for focused mental health interventions. Socio-economic determinants, cultural norms, and challenges related to healthcare infrastructure are likely contributing factors to the high prevalence rates. Addressing these challenges requires developing strategies to ensure adequate mental health services and support systems in densely populated and high-risk areas.

Another study [21] highlighted that living in Java is a significant contributing factor to depression among older adults in Indonesia. This helps explain why the highest prevalence of depression is seen in the provinces on Java Island. On the other hand, the findings of this study differ slightly from those reported previously [22], which showed that Jambi and Central Sulawesi had the lowest and highest depression rates, respectively. This difference may indicate progress in governmental efforts to reduce depression, though further exploration of local context and intervention efficacy is needed.

In Indonesia, the government has shifted focus toward promotive and preventive approaches to mental health [23]. According to the Director General of Public Health, Maria Endang Sumiwi, mental health programs in the country are transitioning from curative and rehabilitative measures to more proactive strategies. This includes increasing early detection, improving case management, and raising awareness at the primary care level. Additionally, the government has been working on developing a mental immunization program through the P2MKJN Directorate [12], which seeks to build resilience and reduce the burden of mental health issues. Apart from that, to facilitate access to digital health services, the Ministry of Health is developing mental health applications as has been proposed earlier [24] and the effectiveness has been discussed and highlighted in previous studies [25-34].

In addition to government policies, the prevalence of depression can also be influenced by several other factors. Other studies [35] suggest that various factors, including age, gender, marital status, physical activity, and smoking, affect depression rates in Indonesia. In particular, efforts to address these related risk factors could reduce the prevalence of depression across the country. For example, reducing smoking, encouraging physical activity, and preventing chronic illnesses such as hypertension and cancer could play a significant role in mitigating depression.

The 2023 SKI results further demonstrate that depression prevalence is slightly higher in males than in females. Additionally, urban residents are more likely to suffer from depression compared to rural populations. People from higher socio-economic backgrounds, as well as those in the 15-54 age range, show a higher prevalence of depression. Conversely, rural residents, individuals with lower economic status, and certain other demographic groups, such as the elderly (75+), show lower depression rates.

These findings differ from those presented in the Riskesdas 2018 survey [11], particularly in terms of gender and age. Additionally, when compared to studies from other low- and middle-income countries, such as those in India, Pakistan, Sub-Saharan Africa, and Latin America, it becomes evident that depression prevalence is generally higher among women and in urban areas. For example, studies in countries like India and Nigeria have shown higher depression rates in rural areas, in contrast to the findings in Indonesia, as discussed in a previous study [36-43]. These differences suggest that while urbanization tends to increase depression rates in Indonesia, in some other regions, rural areas experience greater mental health challenges due to factors such as limited healthcare access.

These comparisons help identify Indonesia's unique challenges in addressing mental health issues, particularly in urbanized and densely populated areas like Jawa Barat, Jawa Tengah, and Jawa Timur. One critical takeaway is that public health strategies must be tailored to the specific socio-economic and cultural contexts of each region. For example, rural areas in Indonesia may require different interventions from urban areas, such as mobile clinics or telemedicine services, to overcome access barriers.

In light of these findings, the Indonesian government should exercise caution when implementing policies to address depression prevalence, considering the distinct needs of both rural and urban populations. By focusing resources on areas with high depression rates, especially in Jawa Barat, Jawa Tengah, and Jawa Timur, policymakers can develop more effective and targeted interventions. Mental health immunization programs and the Sehat Jiwa application should be implemented more intensively in regions with high prevalence clusters, including these provinces. Furthermore, enhancing early detection, prevention, and case management services at the primary care level will be critical to improving mental health outcomes nationwide.

This clustering study offers a solid scientific basis for planning and evaluating mental health policies and reinforces the Ministry of Health’s ongoing mental health programs. The findings of this research can help direct resources more efficiently, ensuring that interventions reach those most in need, ultimately contributing to better mental health outcomes across Indonesia.

## CONCLUSION

The analysis of depression prevalence over the past two weeks among individuals aged ≥15 years by province in Indonesia, using the Natural Breaks (Jenks) method in RStudio, revealed distinct regional disparities. The dataset was categorized into four clusters: low, medium, high, and very high prevalence. The results indicate a clear geographical pattern, with provinces showing lower prevalence rates clustered together while those with higher prevalence rates are concentrated in specific areas. This suggests that regional factors, such as socio-economic conditions, access to mental health services, cultural differences, and environmental stressors, may influence depression prevalence. Overall, Indonesia has more provinces with low depression prevalence than those with high rates. Understanding this distribution can help policymakers and healthcare professionals design targeted interventions and allocate resources more effectively based on regional needs. By focusing on areas with higher prevalence rates, interventions can be better tailored to reduce the burden of depression and improve mental health outcomes across the country. These findings indicate that the government should prioritize resource allocation in provinces such as Jawa Barat, Jawa Tengah, and Jawa Timur to enhance mental health outcomes nationwide.

## Figures and Tables

**Fig. (1):**
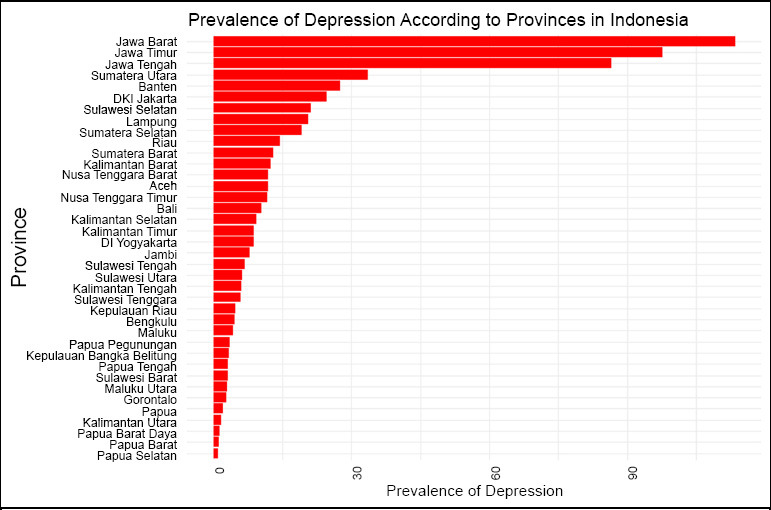
Bar plot prevalence of depression.

**Fig. (2):**
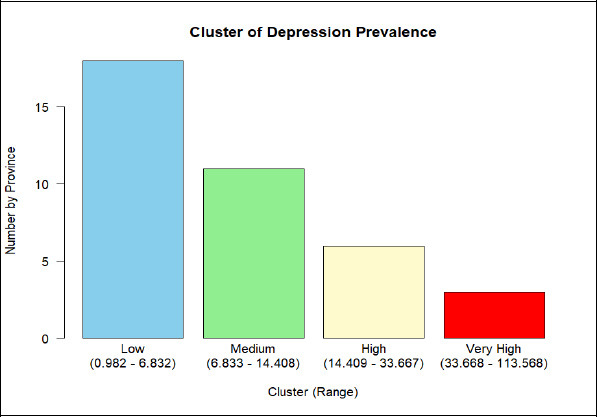
Bar plot number of provinces by cluster.

**Fig. (3):**
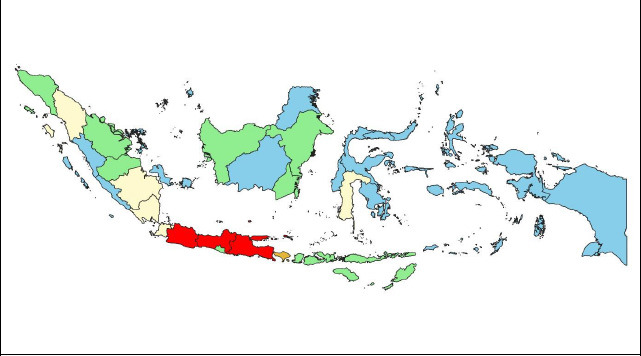
Mapping cluster of depression prevalence in indonesia.

**Table 1:** Prevalence of depression by provinces in indonesia [14].

Province	Prevalence	Province	Prevalence	Province	Prevalence
Aceh	11,858	DI Yogyakarta	8,827	Sulawesi Selatan	21,208
Sumatera Utara	33,667	Jawa Timur	97,746	Sulawesi Tenggara	5,912
Sumatera Barat	12,973	Banten	27,507	Gorontalo	2,753
Riau	14,408	Bali	10,412	Sulawesi Barat	3,171
Jambi	7,890	Nusa Tenggara Barat	11,964	Maluku	4,273
Sumatera Selatan	19,282	Nusa Tenggara Timur	11,779	Maluku Utara	2,920
Bengkulu	4,674	Kalimantan Barat	12,525	Papua Barat	1,219
Lampung	20,646	Kalimantan Tengah	6,163	Papua Barat Daya	1,359
Kepulauan Bangka Belitung	3,439	Kalimantan Selatan	9,301	Papua	2,059
Kepulauan Riau	4,778	Kalimantan Timur	8,850	Papua Selatan	0,982
DKI Jakarta	24,697	Kalimantan Utara	1,637	Papua Tengah	3,249
Jawa Barat	113,568	Sulawesi Utara	6,178	Papua Pegunungan	3,454
Jawa Tengah	86,668	Sulawesi Tengah	6,832		

**Table 2:** The results of depression prevalence clusters by natural jenks method.

Cluster	Depression Prevalence	Number of Provinces	Mean	Median	Standard Deviation
Low	0,982 – 6,832	18	3,614	3,344	1,836
Medium	6,833 – 14,408	11	10,981	11,799	2,058
High	14,409 – 33,667	6	24,502	22,952	5,404
Very High	33,668 – 113,568	3	99,327	97,746	13,519

## Data Availability

The data supporting the findings of the article is available at http://bit.ly/43yBgVz.

## LIST OF ABBREVIATIONS

WHO: = World Health Organization
ICD: = International Classification of Diseases
GIS: = Geographic Information System
MINI: = Mini International Neuropsychiatric Interview
CB: = Census Blocks

## STANDARDS OF REPORTING

STROBE guidelines were followed.
